# Treatment of prolonged drug reaction with eosinophilia and systemic symptoms syndrome with dupilumab using a molecularly-guided approach

**DOI:** 10.1016/j.jdcr.2024.03.020

**Published:** 2024-04-09

**Authors:** Kailyn Valido, Vandan Patel, Michael J. Murphy, Muhammad H. Junejo, Devisha K. Patel, Alana Deutsch, Noel Turner, Theodore D. Zaki, Brett King, William Damsky, Caroline A. Nelson

**Affiliations:** a Department of Dermatology, Yale School of Medicine, New Haven, Connecticut; b Department of Dermatology, Hackensack Meridian Health Palisades Medical Center, North Bergen, New Jersey; c Department of Internal Medicine, Northwell Health Lenox Hill Hospital, New York, New York; d Department of Pathology, Yale School of Medicine, New Haven, Connecticut

**Keywords:** DRESS, drug reaction with eosinophilia and systemic symptoms, dupilumab, leflunomide, RNA in situ hybridization

## Introduction

Drug reaction with eosinophilia and systemic symptoms (DRESS) syndrome is a severe cutaneous adverse reaction characterized by an exanthem, fever, and hematologic and visceral organ involvement^1^ that often requires prolonged high-dose systemic corticosteroid therapy. Targeted steroid-sparing therapies may be beneficial, particularly for patients with steroid-dependent or steroid-refractory DRESS or those at increased risk of corticosteroid-related adverse effects. Here we describe a case of prolonged DRESS in which skin molecular profiling via RNA in situ hybridization (RISH) guided the selection of dupilumab as an effective targeted steroid-sparing therapy.

## Case report

A 52-year-old female with hypothyroidism and an autoimmune diathesis with features of Sjögren’s and antisynthetase syndrome developed a generalized rash and abdominal pain 4 weeks after starting leflunomide (20 mg daily) for arthritis. After self-discontinuation of leflunomide, she was started on hydroxychloroquine (200 mg daily) and a prednisone taper of 30 mg daily by her rheumatologist. Two weeks later, she presented to the emergency department for persistence of the rash and abdominal pain. Her medications at the time of presentation included levothyroxine, hydroxychloroquine, prednisone (10 mg daily), and intermittent ibuprofen.

Physical examination revealed pink blanching macules and papules on the face, trunk, and extremities with associated facial edema. Skin biopsy demonstrated interface change, spongiosis, and a mixed inflammatory infiltrate, compatible with a drug reaction. Laboratory results were notable for eosinophilia with an absolute eosinophil count of 0.96 × 10^9^/L (peak: 1.56 × 10^9^/L) and scattered atypical lymphocytes on peripheral blood smear. Further investigation revealed an elevated lipase of 1960 U/L (peak value) and transaminitis with an alanine aminotransferase of 279 U/L (peak: 349 U/L) and aspartate aminotransferase of 214 U/L (peak: 263 U/L). Serologies for Epstein-Barr virus, cytomegalovirus (CMV), and human herpes virus 6 were notable for past infection. A chest computed tomography scan showed interstitial lung disease and an abdominal computed tomography revealed acute pancreatitis. The patient was diagnosed with DRESS suspected secondary to leflunomide with a RegiSCAR score of 7, corresponding to “definite DRESS”.^1^

**Fig 1:**
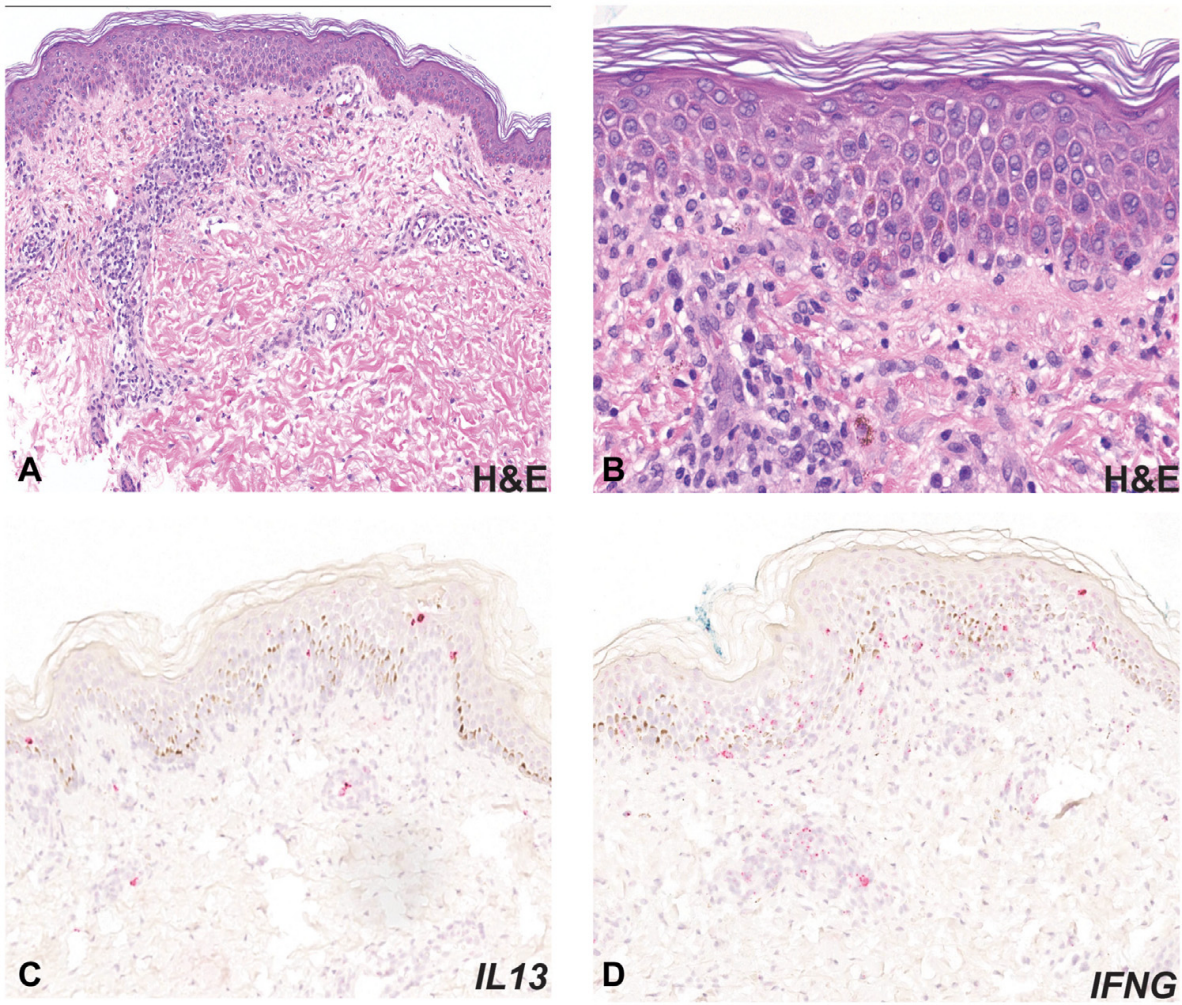
Histology and cytokine staining. Skin biopsy specimen of the left thigh revealing spongiosis, focal vacuolar change, and a mixed inflammatory infiltrate. (Hematoxylin and eosin, 100× magnification (**A**), 200× magnification (**B**)). mRNA in situ hybridization (RISH) staining (*red* chromogen) for IL13 (**C**) and IFNG (**D**). *H&E*, Hematoxylin and eosin; *IFNG*, interferon gamma; *IL13*, interleukin 13.

**Fig 2:**
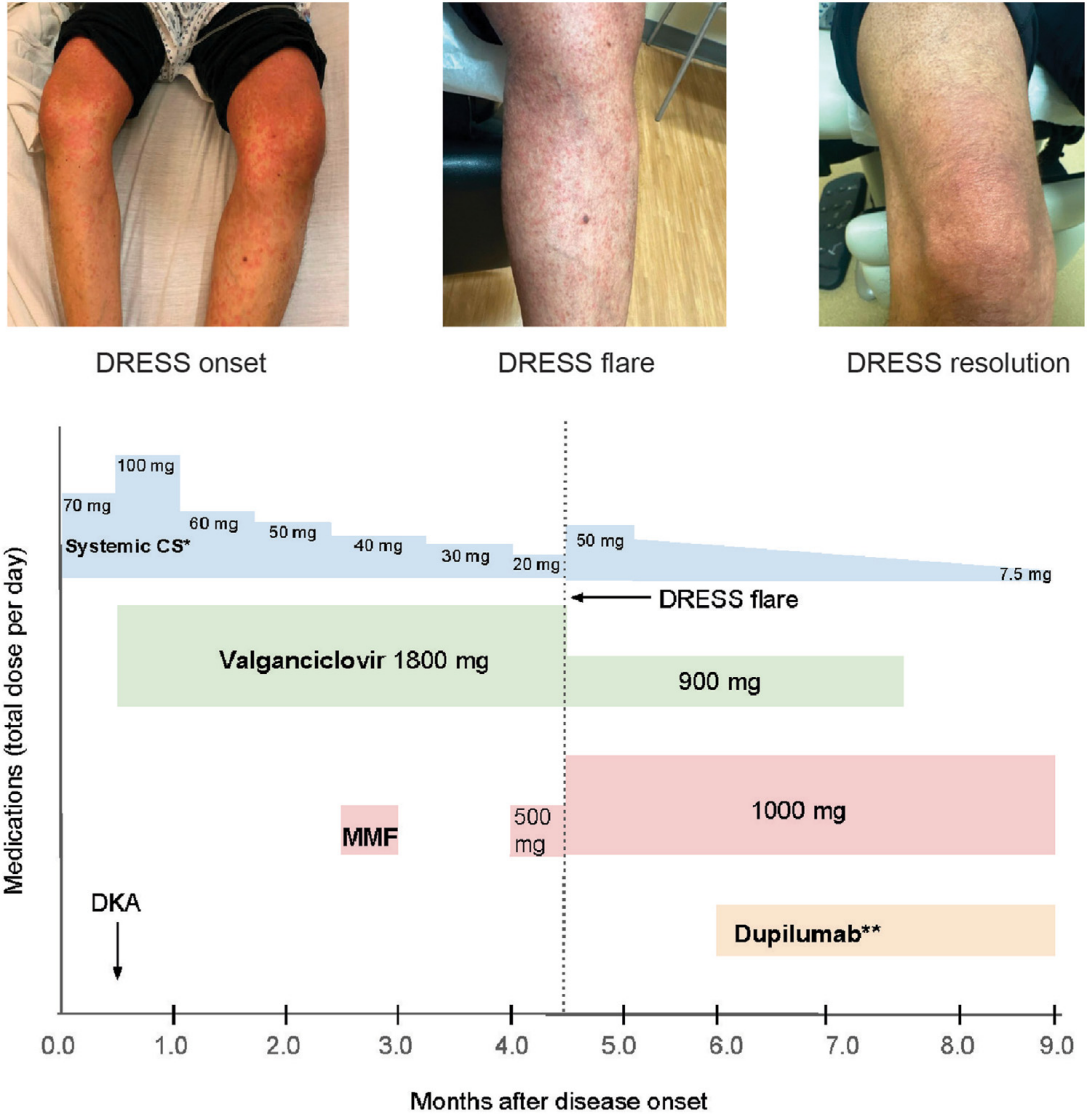
Treatment timeline. Treatment timeline and clinical photos of our patient with prolonged, steroid-dependent DRESS. *CS*, Corticosteroids; *DKA*, diabetic ketoacidosis; *DRESS*, drug reaction with eosinophilia and systemic symptoms; *MMF*, mycophenolate mofetil. ∗Prednisone equivalent dosing. ∗∗Dupilumab dosing: initial loading dose of 600 mg (2 × 300 mg), followed by 300 mg every 2 weeks.

The patient was initiated on prednisone 70 mg daily (1 mg/kg) and topical corticosteroids, which led to clinical improvement and down-trending of the eosinophil count and liver enzymes. One week after discharge, she was readmitted with diabetic ketoacidosis thought to be secondary to DRESS and high dose corticosteroid use in the setting of recent pancreatic injury. Laboratory results revealed repeated transaminitis with an alanine aminotransferase of 181 U/L (peak: 469 U/L), aspartate aminotransferase of 88 U/L (peak: 326 U/L), and total bilirubin of 2.7 mg/dL (peak: 5.9 mg/dL). Workup also demonstrated CMV viremia (9745 copies/mL) and a C-peptide of 0.10 ng/mL (normal: 0.80-3.85 ng/mL). Islet cell antibody screen and glutamic acid decarboxylase antibody were negative. Her diabetic ketoacidosis was managed in the intensive care unit, and the CMV hepatitis improved on methylprednisolone 80 mg daily and valganciclovir (900 mg twice daily). The patient was discharged on prednisone 60 mg daily, valganciclovir, and insulin therapy. Soon after, mycophenolate (250 mg twice daily) was initiated for her autoimmune condition. Due to a known breast lesion on prior imaging, workup revealed ductal carcinoma in situ, which was treated with lumpectomy, radiation, and anastrozole. Approximately 3 months later in the setting of tapering prednisone to 20 mg daily and remaining off leflunomide, hydroxychloroquine, and ibuprofen, the patient redeveloped a pruritic rash covering 70% body surface area. Repeat biopsy demonstrated spongiosis and focal vacuolization of the dermoepidermal junction (Fig 1, *A* and *B*), and prednisone was increased (50 mg daily). Given the eruption in the setting of prednisone taper, RISH was performed on the initial and repeat biopsies, revealing strong expression of interleukin (IL)4, IL13, and interferon-gamma in both (Fig 1, *C* and *D*). There was no significant staining for CD123 or myxovirus resistant protein A, which are markers for type I interferon activation typical of connective tissue diseases. The rash, diagnosed as steroid-dependent DRESS, resolved with dupilumab and remained clear despite tapering prednisone to 7.5 mg daily, as illustrated in the treatment timeline (Fig 2).

## Discussion

In this report, we describe a case of prolonged DRESS syndrome. According to the Naranjo Algorithm as well as the timing of the patient’s presentation with respect to the drug, leflunomide was considered the most likely drug culprit with a score of 6 (probable) versus less likely hydroxychloroquine with a score of 1 (possible).^2^ Though less common than other drugs, leflunomide as an offending agent in DRESS has been described in the literature.^3^, ^4^, ^5^, ^6^, ^7^, ^8^, ^9^, ^10^, ^11^, ^12^, ^13^ Selected clinical characteristics of these cases are shown in Table I.

**Table I:** Reported cases of leflunomide-induced drug reaction with eosinophilia and systemic symptoms syndrome: Selected clinical characteristics, treatment, and outcomes

Case∗	Current case	Uppal et al^3^	Shastri et al^4^	Shastri et al^4^	Shastri et al^4^	Shastri et al^4^	Shastri et al^4^	Do-Pham et al^5^	Vaish et al^6^	Parajuli et al^7^	Pinto et al^8^	Rao et al^9^	Gayfield et al^10^	Fatima et al^11^
Age (years), sex	52, F	57, F	21, F	24, M	50, F	52, M	47, F	70, F	40, M	40, F	50, M	52, M	35, M	32, F
Leflunomide indication	Arthritis	RA	Arthralgia	Arthritis	Arthritis	RA	RA	Psoriatic arthritis	Joint pains	RA	Knee pain	RA	RA	Arthritis
Latency period (weeks)	4	12	6	4	5	6	2	4	3	1	4	1	4	NR†
RegiSCAR criteria (all variables as defined by the RegiSCAR scoring system^1^)‡														
Fever	No (−1)	Yes (0)	Yes (0)	Yes (0)	Yes (0)	Yes (0)	Yes (0)	Yes (0)	Yes (0)	Yes (0)	Yes (0)	Yes (0)	No (−1)	Yes (0)
LAD	No (0)	Yes (1)	NR (0)	NR (0)	Yes (1)	NR (0)	No (0)	Yes (1)	No (0)	Yes (1)	Unknown (0)	Yes (1)	No (0)	No (0)
Eosinophilia	Yes (2)	NR (0)	No (0)	Yes (2)	No (0)	No (0)	Yes (2)	NR (0)	Yes (2)	Yes (1)	Yes (1)	Yes (2)	No (0)	Yes (2)
Atypical lymphocytes	Yes (1)	NR (0)	NR (0)	NR (0)	NR (0)	NR (0)	NR (0)	NR (0)	NR (0)	Yes (1)	Yes (1)	Yes (1)	No (0)	Yes (1)
≥50% BSA	Yes (1)	Yes (1)	Yes (1)	Yes (1)	Yes (1)	Yes (1)	Yes (1)	NR (0)	Yes (1)	Yes (1)	Yes (1)	Yes (1)	Yes (1)	Yes (1)
≥2 of: edema, infiltration, purpura, scaling	Yes (1)	Yes (1)	Yes (1)	Yes (1)	Yes (1)	Yes (1)	NR (0)	NR (0)	Yes (1)	Yes (1)	Yes (1)	NR (0)	Yes (1)	Yes (1)
Biopsy suggesting DRESS	Yes (0)	Yes (0)	NR (0)	NR (0)	Yes (0)	Yes (0)	Yes (0)	NR (0)	Unknown (0)	Yes (0)	Unknown (0)	Yes (0)	No (−1)	Unknown (0)
Organ involvement	Liver, pancreas (2)	Liver (1)	Liver (1)	No (0)	Liver (1)	No (0)	Liver (1)	Liver, kidney, GI tract (2)	Liver (1)	Liver, kidney (2)	Liver, kidney, GI tract (2)	No (0)	Liver (1)	Liver (1)
Rash resolution ≥15 days	Yes (0)	Yes (0)	Yes (0)	Yes (0)	Yes (0)	NR (0)	NR (0)	NR (0)	Yes (0)	Yes (0)	Yes (0)	No (−1)	Yes (0)	Yes (0)
Alternative diagnoses excluded	Yes (1)	NR (0)	NR (0)	NR (0)	NR (0)	NR (0)	NR (0)	Unknown (0)	Unknown (0)	NR (0)	Yes (1)	NR (0)	Yes (1)	Yes (1)
RegiSCAR	7 (documented)	4	3	4	4	2	4	6 (documented)	5	7	6 (documented)	4	2 (documented)	7 (documented)
Treatment	Topical CS, systemic CS, MMF, dupilumab	Supportive care	Systemic CS	Systemic CS	Systemic CS	Systemic CS	Supportive care	Supportive care	Systemic CS	Systemic CS	Systemic CS	Systemic CS	Systemic CS	Systemic CS
Outcome	Recovery	Death from HE	Death from TEN	Recovery	NR	Recovery	Lost to follow-up	Death from GI hemorrhage	Recovery	Death from high-output HF	Death from perforation peritonitis	Recovery	Recovery	Death from unspecified cause

*BSA*, Body surface area; *CS*, corticosteroids; *GI*, gastrointestinal; *HE*, hepatic encephalopathy; *HF*, heart failure; *LAD*, lymphadenopathy; *MMF*, mycophenolate mofetil; *NR*, not reported; *RA*, rheumatoid arthritis; *TEN*, toxic epidermal necrolysis.

∗ Sultan et al^12^ and Devarbhavi et al^13^ reported 1 case and 6 cases of leflunomide-induced DRESS syndrome, respectively, but pertinent individual-level clinical data is not available.

† The exact latency period is not specified, but the article states that DRESS symptoms began “a few weeks” after leflunomide intake.

‡ If RegiSCAR score was not documented in the article, a minimum RegiSCAR score was calculated based on data reported in each article.

This case of DRESS was steroid-dependent and complicated by multiorgan involvement and CMV reactivation. Though our patient improved on high-dose systemic corticosteroids, cutaneous symptoms returned when her prednisone dose was tapered to 20 mg daily. This prompted consideration of steroid-sparing alternatives, as prolonged systemic corticosteroid therapy is associated with a morbid adverse event profile. We performed cytokine staining via RISH, a highly specific RNA staining technique that has been increasingly utilized in obtaining individual molecular phenotypes of different dermatologic conditions.^14^ Based on the patient’s RISH results, dupilumab was selected as an adjuvant treatment while the prednisone was tapered. Janus kinase (JAK) inhibitor therapy, which has been reported in DRESS,^15^ was also considered as an alternative steroid-sparing agent due to its inhibition of IL-4, IL-13, and interferon-gamma. In this patient, dupilumab led to rapid resolution of her pruritic eruption despite tapering prednisone to a physiologic dose, suggesting that type 2 inflammation was the primary driver of her pruritic rash.

Dupilumab, a monoclonal antibody against IL-4Rα, inhibits signaling of IL-4 and IL-13. Research shows that there are higher levels of circulating IL-4 and IL-13 producing CD4+ T-cells in patients with active DRESS compared to healthy controls, and these levels decline during recovery.^16^ There are 3 prior reports of DRESS treated with dupilumab.^17^^,^^18^ All cases were steroid-dependent or steroid-refractory, prompting the selection of dupilumab and ultimately leading to recovery. One case noted elevated CD4+ T cells secreting IL-4 and IL-13 in the serum, supporting the use of dupilumab.^18^ To our knowledge, this is the first report in which molecular profiling of the skin via RISH was utilized to guide the selection of dupilumab as an effective targeted steroid-sparing therapy for DRESS. This report supports the use of dupilumab in steroid-dependent or steroid-refractory cases of DRESS and illustrates a novel application of precision medicine in dermatology.

## Conflicts of interest

Dr Damsky is a consultant for Pfizer, Incyte, Eli Lilly, and TWi Biotechnology, and has research funding from Pfizer, Advanced Cell Diagnostics/Bio-techne, and AbbVie and receives licensing fees from EMD/Millipore/Sigma, all outside the submitted work. Dr Nelson has received research grants from Boehringer Ingelheim and participated in an advisory board for work related to pustular psoriasis and palmoplantar pustulosis. All other authors have no relevant conflicts to disclose.
